# The associations between p,p’-DDE levels and plasma levels of lipoproteins and their subclasses in an elderly population determined by analysis of lipoprotein content

**DOI:** 10.1186/s12944-020-01417-1

**Published:** 2020-12-07

**Authors:** Juliann Jugan, P. Monica Lind, Samira Salihovic, Jordan Stubleski, Anna Kärrman, Lars Lind, Michele A. La Merrill

**Affiliations:** 1 Department of Environmental Toxicology, University of California, One Shields Avenue, Davis, CA 95616 USA; 2 Department of Medical Sciences, Occupational and Environmental Medicine, Uppsala University, Uppsala, Sweden; 3 School of Medical Sciences, Inflammatory Response and Infection Susceptibility Centre, Örebro University, Örebro, Sweden; 4 MTM Research Centre, School of Science and Technology, Örebro University, Örebro, Sweden; 5 Wellington Laboratories Inc., Guelph, ON Canada; 6 Department of Medical Sciences, Cardiovascular Epidemiology, Uppsala University, Uppsala, Sweden

**Keywords:** Lipoprotein, Triglycerides, Dichlorodiphenyldichloroethylene, Persistent organic pollutants, Cholesterol, Cardiovascular disease, Phospholipids

## Abstract

**Background:**

Lipoproteins at aberrant levels are known to play a role in cardiovascular disease. The metabolite of the insecticide dichlorodiphenyltrichloroethane (DDT), p,p’-dichlorodiphenyldichloroethylene (p,p’-DDE), physically associates with lipids and accumulates in adipose tissue. Little is known about which lipoproteins associate with p,p’-DDE. An association between p,p’-DDE exposure and altered levels of circulating lipids was assessed in a large human cohort using a detailed analysis of lipoprotein content.

**Methods:**

Plasma samples were collected from the subset of 75-year old Swedes in the Prospective Investigation of the Vasculature of Uppsala Seniors (PIVUS) cohort who were not prescribed lipid lowering medication (*n* = 571). p,p’-DDE concentrations in plasma were measured using high-throughput solid phase extraction and gas chromatography-high resolution mass spectrometry. Analysis of plasma lipoprotein content was performed with nuclear magnetic resonance spectroscopy.

**Results:**

Detectable levels of p,p’-DDE were found in the plasma samples of all subjects. Elevated p,p’-DDE levels were associated with increased concentrations of lipoproteins of all diameters, with the exception of high density lipoprotein (HDL) of diameters between 14.3 nm–10.9 nm. Of the lipoprotein constituents, triglycerides were most uniformly associated with elevated p,p’-DDE across lipoproteins. p,p’-DDE was furthermore associated with apolipoprotein B, but not apolipoprotein A1.

**Conclusions:**

The positive associations observed between each lipoprotein class and elevated p,p’-DDE support previous data suggesting that p,p’-DDE interacts with lipoproteins within plasma. It is speculated that both physio-chemical and biological mechanisms may explain why p,p’-DDE does not uniformly associate with lipids across lipoproteins.

**Supplementary Information:**

The online version contains supplementary material available at 10.1186/s12944-020-01417-1.

## Introduction

Cardiovascular disease (CVD) is the primary cause for mortality in adults age 35–75 regardless of nationality or income [1]. Increased circulating levels of triglycerides, cholesterol, and phosphatidylcholines have been associated with an elevated risk of CVD in lipidomic analyses of human plasma in multiple population-based cohorts [2, 3]. Although lipid profiles can be strongly influenced by lifestyle and family history, additional environmental factors are likely to contribute to a risky lipid profile given that CVD permeates global populations [4].

The metabolite of the insecticide dichlorodiphenyltrichloroethane (DDT), p,p’-dichlorodiphenyldichloroethylene (p,p’-DDE), has been associated with increased risk of CVD in multiple human cohorts. Within the Prospective Study of Vasculature in Uppsala Seniors (PIVUS) cohort, p,p’-DDE has been associated with increased hypertension [5, 6] and is considered a predictor of stroke at elevated baseline levels [7]. Through the mediation of obesity, elevated left ventricular mass was associated with p,p’-DDE in the PIVUS cohort, suggesting that metabolic risk factors associated with p,p’-DDE exposure are involved in the relationship between p,p’-DDE and CVD [6]. Increased risk of stroke was also observed in participants with high serum concentration of p,p’-DDE in the Korean Cancer Prevention Study-II, showing that this association is replicated across cohorts [8].

These findings were further replicated in rodent models with doses of p,p’-DDT or p,p’-DDE relevant to human exposure levels. In mice, perinatal exposure to p,p’-DDT induced hypertension and cardiac hypertrophy [9]. Hypertension was also observed in adult Wistar rats dosed with p,p’-DDE, regardless of diet [10]. It is likely that this interaction with the cardiovascular system is mediated through lipid metabolism, as multiple rodent models have confirmed p,p’-DDT exposures lead to elevated lipids in both the blood and liver [11]. It is important to note that other factors, such as temporality, did not confound conclusions in these models [9–11].

Despite the restricted use and production of DDT by the United Nations Stockholm Convention, the health risk of this chemical is still substantial due to continued exposure [12]. Exposure risk is greatest in nations that have yet to ratify the Stockholm Convention or in those that continue to use DDT for control of the malaria vector [13–15]. Worldwide exposure to p,p’-DDE occurs due to bioaccumulation of this persistent organic pollutant (POP) in the fat of animals that make up our diet [16]. Pervasive exposure to p,p’-DDE could play a role in the risk profile for CVD, contributing to the worldwide risk of mortality due to this disease. An example of this link has previously been demonstrated in an elderly cohort, where individuals with high serum organochlorine pesticides, inclusive of p,p’-DDE, and low fat mass showed a 4.5 times greater risk of CVD mortality [17].

Lipid dysfunction is a common pathology among a significant portion of the risk factors for CVD, such as diabetes, obesity, hyperlipidemia, and hypertension [18, 19]. Together, this cluster of CVD risk factors is considered metabolic syndrome, which has been positively associated with high plasma concentrations of p,p’-DDE [20]. Numerous epidemiological studies show that p,p’-DDE is found within human plasma and adipose tissue due to its high lipophilicity [21], however, it is unclear which lipids p,p’-DDE associates with to facilitate its activity [22, 23]. These characteristics of high persistence allow p,p’-DDE to affect adipose tissue and lipid metabolism while it is stored within the body for an estimated half-life of 6.2–8.9 years [21, 24].

Although both high and low density lipoproteins have been shown to associate with p,p’-DDE in human plasma, conclusions on which lipoproteins p,p’-DDE associates most strongly with were discordant between studies [25, 26]. While plasma from fasted adults showed the strongest association between p,p’-DDE and low density lipoprotein (LDL) [25], plasma from non-fasted adults showed the greatest association between p,p’-DDE and both very low density lipoprotein (VLDL) and LDL [26]. Neither study assessed intermediate density lipoprotein (IDL), nor did they measure associations between p,p’-DDE and the lipid constituents that made up each lipoprotein class. The present study aimed to use a detailed analysis of lipoprotein content to quantify the association between p,p’-DDE and blood lipids. This method allows for the inclusion of lipids and lipoproteins not reflected by routine clinical lipid parameters, as well as the differentiation between lipoprotein sizes.

## Methods

### Participants

The study sample is a subset of the PIVUS cohort. Elderly participants residing in Uppsala, Sweden were eligible and received an invitation to participate in the study 2 months before their 70th birthday. Invitation order was randomized, and of the 2025 community members invited, 1016 chose to participate (50.1%). Between April 2001 and June 2004, participants were medically examined and given a questionnaire regarding their medical history, lifestyle, and regular medication [27]. Subjects were invited for follow-up at age 75 and 80. From the follow-up at age 75 with 826 participants, 571 participants free from lipid-lowering medication were selected for lipoprotein analysis between 2006 and 2009.

Written and informed consent was obtained from all participants by the University of Uppsala. The protocol was approved by the Ethics Committee of the University of Uppsala, and complies with the Declaration of Helsinki.

### Blood sampling

Blood samples were collected from all participants in the morning (8-10 am) following an overnight fast using EDTA-coated plasma glass tubes. During the fast, no medication or smoking was allowed after midnight. Plasma was separated from the blood and stored in Eppendorf (Horsholm, Denmark) tubes at − 80 °C for later analysis.

### Lipoprotein content analysis

High-throughput proton nuclear magnetic resonance (^1^H NMR) spectroscopy was used to assess the lipoprotein profile in each 350 μL plasma sample (Nightingale Health Ltd., Helsinki, Finland). The complete protocol can be found in published methods papers [28, 29]. Briefly, plasma samples were diluted in sodium phosphate buffer (75 mmol/L Na_2_HPO_4_ in 80%/20% H_2_O/D_2_O, pH 7.4, 0.08% sodium 3-(trimethylsilyl)propionate-2,2,3,3-d_4_, and 0.04% sodium azide). Liquid was handled with a PerkinElmer JANUS Automated Workstation with an 8-tip dispense arm with Varispan. Lipoprotein measurements were automatically collected with a combined spectrometer system of a Bruker AVANCE III 500 MHz and a Bruker AVANCE III HD 600 MHz. Samples were then manually extracted for lipids using a standardized procedure with multiple extraction steps containing saturated sodium chloride solution, methanol, dichloromethane, and deuterochloroform. Lipid measurements were automatically collected with the Bruker AVANCE III HD 600 MHz. Quality control (QC) samples included a serum mimic and a mixture of two low-molecular weight metabolites. The initial data processing was performed on the spectrometer computers, from which the spectra were then collected and further spectral processing steps were performed including: overall signal check, background control, spectral area-specific signal alignments, and baseline removal. Quantified molecular data underwent QC for statistical quality and was compared to a database of quantitative molecular data [28]. Subclasses of lipoproteins were defined as follows: five very large lipoprotein (VLDL) subclasses between 64 nm – 31.3 nm in diameter, intermediate density lipoprotein (IDL) at a diameter of 28.6 nm, three low density lipoprotein (LDL) subclasses between 25.5 nm – 18.7 nm in diameter, and four high density lipoprotein (HDL) subclasses between 14.3 nm – 8.7 nm in diameter. The subclass extremely large VLDL, with particle diameter greater than 75 nm, was not included in the present study because measurements were above the level of detection. Lipoprotein components including: cholesterol esters (CE), free cholesterol (FC), triglycerides (TG), phospholipids (PL), total cholesterol (C), and total lipids (L) were included in the quantification.

### Analysis of p,p’-DDE

The sample preparation method used for extraction of p,p’-DDE from plasma was previously described [30] and briefly summarized below. Samples diluted in protein precipitating solutions of 9 mM sulfuric acid (Merck, Darmstadt, Germany) and 20% (vol/vol) acetonitrile (Fisher Scientific, Leicestershire, UK) were transferred to an Oasis HLB 96-well plate (Waters Corporation, Milford, Massachusetts, USA). A 40% (vol/vol) methanol (Honeywell Riedel-de Haën, Steinheim, Germany) solution in High-performance liquid chromatography (HPLC)-grade water (Fisher Scientific, Leicestershire, UK) was used to rinse the plate before the plate was dried and p,p’-DDE was eluted with a 1:1 dichloromethane:hexane (Honeywell Riedel-de Haën, Steinheim, Germany and Merck, Darmstadt, Germany respectively) solution. Sulfuric acid modified silica (Sigma Aldrich/Supelco, Steinheim, Germany) and sodium sulfate (Sigma Aldrich/Supelco, Steinheim, Germany) were used for lipid degradation and water removal from the sample extracts. Sample extracts were placed in gas chromatography vials and evaporated overnight with 20 μL tetradecane. Splitless injection was used to inject 2 μL of the final extract of sample onto a DB-5MS capillary column (Agilent Technologies, Santa Clara, California, USA). Instrumental analysis was performed with a gas chromatograph (Agilent Technologies) coupled to a high resolution magnetic sector mass spectrometer (Micromass Autospec Ultima, Waters Corporation, Milford, Massachusetts, USA) operating at ≥10,000 resolving power using electron ionization at 35 eV. Isotope dilution methodology was used with a ^13^C-labeled p,p’-DDE standard to ensure accurate quantification of p,p’-DDE. Specifically, the two most abundant ions of the chlorine cluster of the molecular ion (p,p’-DDE) and the molecular ion of the ^13^C-labeled p,p’-DDE standard were monitored. QC samples included 102 replicate HPLC grade water method blanks, instrument blanks, in-house reference plasma, and Standard Reference Material from the National Institute for Standards and Technology (NIST) 1957. QC were analyzed with each batch of samples to ensure optimal instrument and method performance.

### Statistical analysis

p,p’-DDE, and some of the lipids with a skewed distribution were ln-transformed before analyses. The lipids were thereafter transformed to a SD-scale in order to facilitate the comparison between lipids.

One linear regression model was calculated for each lipid per lipoprotein fraction, with the lipid as the dependent variable, and the independent variables p,p’-DDE, sex, and BMI. We note all subjects had the same age. These calculations were performed using STATA14 (Stata Inc., College Station, Texas, USA). Statistical significance was determined by a *P*-value below 0.05. GraphPad Prism for Windows (GraphPad Software, La Jolla, California, USA) (version 8.1.2) was utilized to design the graphical presentation of the data.

## Results

### Participant characteristics

Of the 571 participants in this study, 296 (52%) were women and 275 (48%) were men. The mean body mass index (BMI) was 26.6 kg/m^2^ (SD 4.3) and the mean plasma p,p’-DDE concentration was 2.2 ng/mL (SD 2.1). The limit of detection for p,p’-DDE was 12 pg/mL and the detection rate in serum for p,p’-DDE was 100%, indicating the persistence of and widespread human exposure to this pollutant. The greatest variance in lipoprotein particle content between individuals was within the VLDL subfractions of lipids (Table 1).

**Table 1:** Descriptive characteristics^a^ of p,p’-DDE and lipoproteins in plasma of PIVUS participants (*n* = 571)

Parameter	Median	IQR	
p,p’-DDE	4.78 × 10^− 9^	2.59 × 10^− 9^	9.15 × 10^− 9^
XL VLDL	1.12 × 10^− 12^	4.00 × 10^− 14^	1.85 × 10^− 12^
L VLDL	4.51 × 10^− 12^	2.90 × 10^− 12^	7.68 × 10^− 12^
M VLDL	2.63 × 10^− 11^	2.16 × 10^− 11^	3.37 × 10^− 11^
S VLDL	3.61 × 10^− 11^	2.93 × 10^− 11^	4.36 × 10^− 11^
XS VLDL	5.47 × 10^− 11^	4.77 × 10^− 11^	6.29 × 10^− 11^
Total IDL	2.61 × 10^− 10^	2.31 × 10^− 10^	2.99 × 10^− 10^
L LDL	5.19 × 10^− 10^	4.52 × 10^− 10^	5.93 × 10^− 10^
M LDL	2.32 × 10^− 10^	2.01 × 10^− 10^	2.65 × 10^− 10^
S LDL	1.52 × 10^− 10^	1.37 × 10^− 10^	1.71 × 10^− 10^
XL HDL	2.28 × 10^− 10^	1.79 × 10^− 10^	2.96 × 10^− 10^
L HDL	1.34 × 10^− 9^	9.36 × 10^− 10^	1.83 × 10^− 9^
M HDL	2.72 × 10^− 9^	2.24 × 10^− 9^	3.24 × 10^− 9^
S HDL	7.86 × 10^− 9^	7.12 × 10^− 9^	8.61 × 10^− 9^

^a^Concentration of p,p’-DDE and plasma lipoproteins in M

Abbreviations: *IQR* Inter quartile range, *XL* Extra large, *L* Large, *M* Medium, *S* Small, *XS* Extra Small, *VLDL* Very low density lipoprotein, *IDL* Intermediate density lipoprotein, *LDL* Low density lipoprotein, *HDL* High density lipoprotein

### Lipoprotein content analysis

A detailed analysis of lipoprotein content was performed by ^1^H NMR spectroscopy to investigate the association between p,p’-DDE levels in plasma and fourteen lipoprotein subclasses. All significant correlations between p,p’-DDE and lipoprotein subclass (*P* < 0.05) were in the positive direction.

**Fig. 1:**
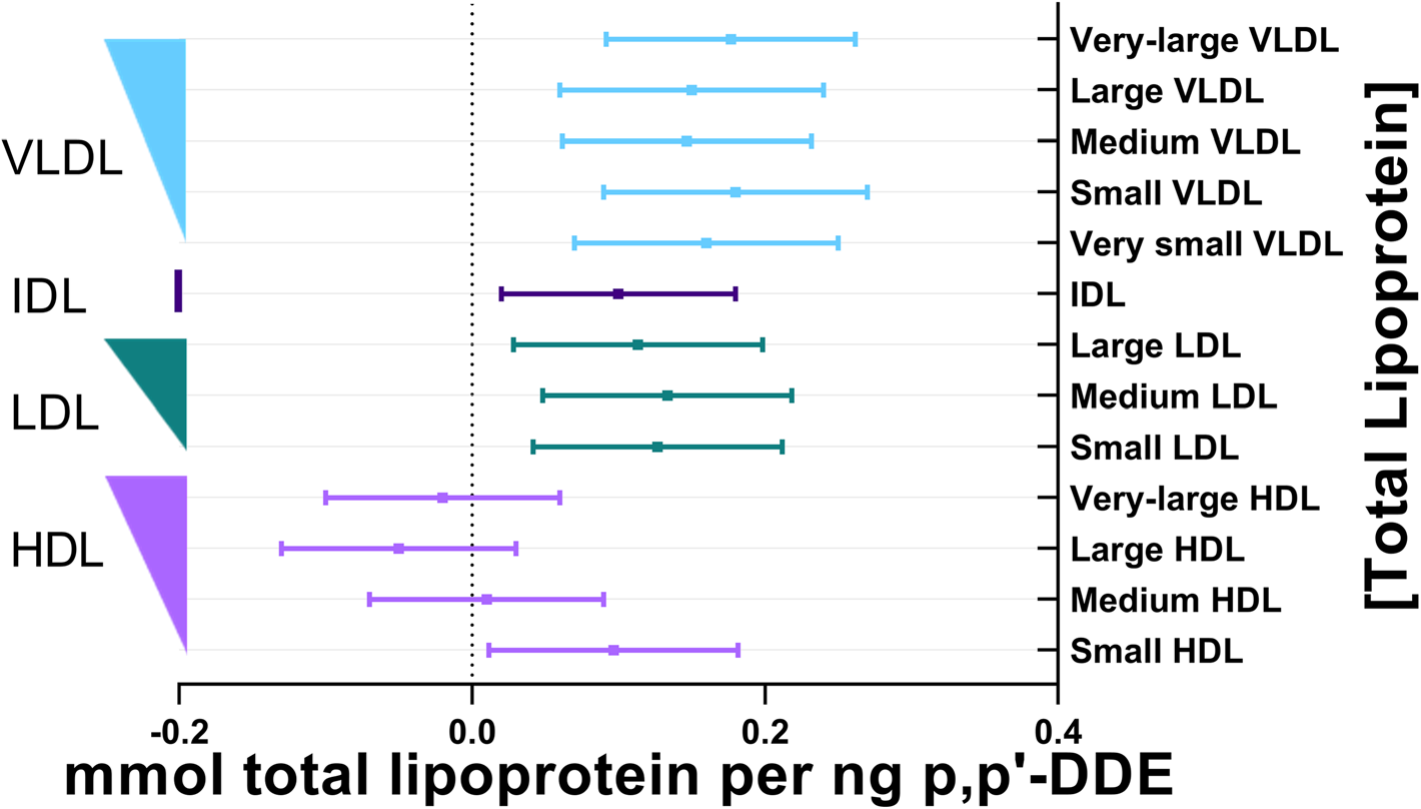
Association between plasma p,p’-DDE (ng) and total lipoprotein of each subclass (mmol). The line of null effect is represented by the x-axis intercept and predicted margins are given together with 95% confidence intervals. The left y-axis illustrates the ordering of lipoprotein diameter in decreasing size, where the width of the right triangle corresponds to the relative diameter of the lipoprotein subclass

The total concentration of all lipoprotein subclasses was significantly elevated in association with p,p’-DDE, with the exception of very-large, large, and medium HDL (Fig. 1). This pattern of associations was mirrored by the association of p,p’-DDE with total lipids, which is inclusive of all lipids measured within each lipoprotein subclass (Supplementary Fig. 1).

**Fig. 2:**
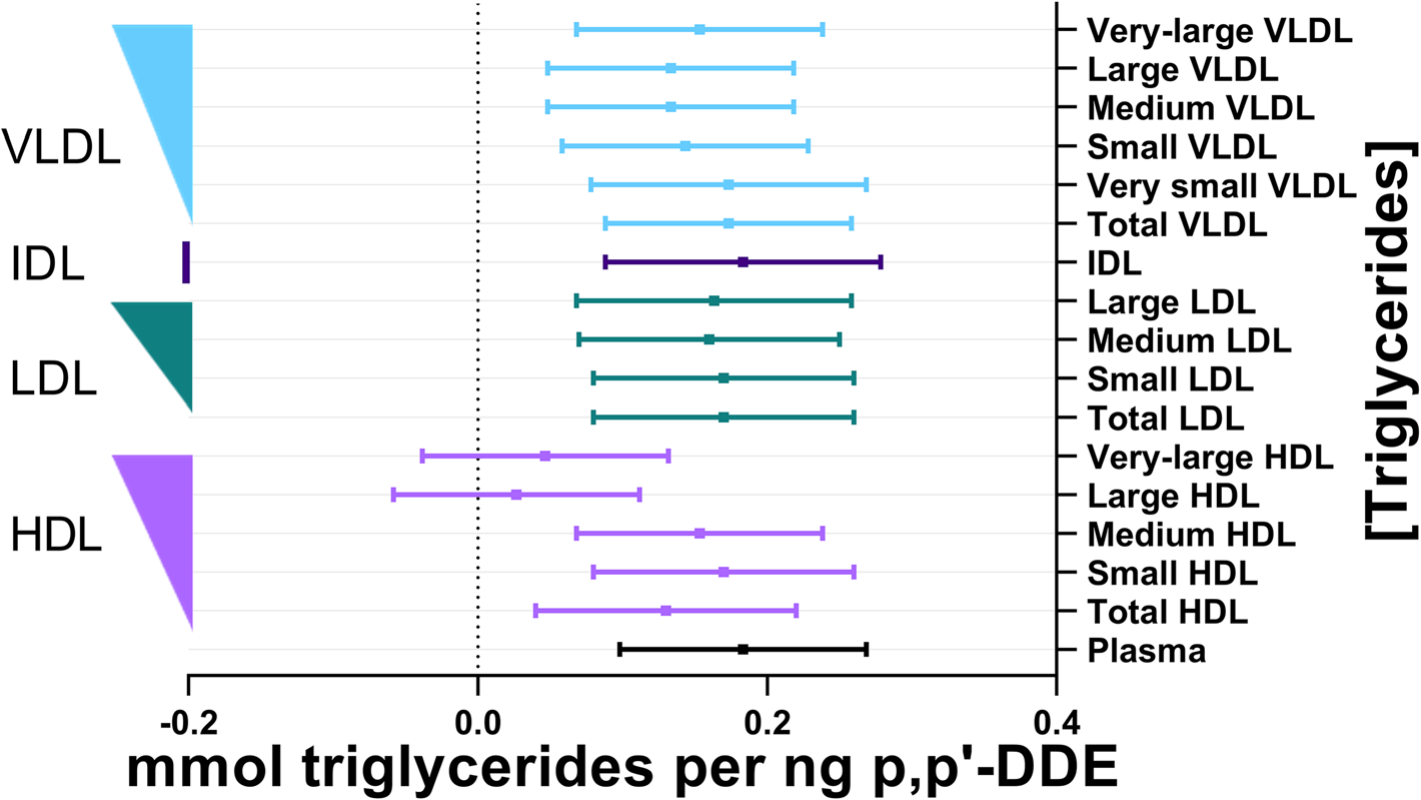
Association between plasma p,p’-DDE (ng) and triglycerides (mmol). The line of null effect is represented by the x-axis intercept and predicted margins are given together with 95% confidence intervals. The left y-axis illustrates the ordering of lipoprotein diameter in decreasing size, where the width of the right triangle corresponds to the relative diameter of the lipoprotein subclass

Among the lipid constituents of the lipoproteins, triglycerides had the most consistent magnitude of significant associations with p,p’-DDE across the lipoprotein subclasses (Fig. 2; Supplementary Table 1). In addition to the expected significant positive association between p,p’-DDE and triglycerides in plasma, the association between p,p’-DDE and triglycerides was also observed among all sizes of VLDL and LDL, in IDL, and in small and medium HDL (Fig. 2). It is possible that the null association of p,p’-DDE and very large HDL was due to a low concentration of triglycerides within very large HDL particles because very large diameter HDL contained the lowest mean concentration of triglycerides (Fig. 2;Supplemental Table 1). However, this hypothesis was not supported by the similar observation that large HDL also had no significant association with p,p’-DDE (Fig. 2;Supplemental Table 1) despite having a greater median percentage of triglycerides than was observed within very large VLDL, medium LDL, and small LDL fractions (Table 2).

**Table 2:** Descriptions^a^ of lipoprotein constituents within each lipoprotein subgroup of PIVUS participants (*n* = 571)

Lipoprotein	Triglycerides			Cholesterol Esters			Free Cholesterol			Total Cholesterol			Phospholipids		
	Median	IQR		Median	IQR		Median	IQR		Median	IQR		Median	IQR	
XL VLDL	2.62	0.00	5.05	3.42	0.00	4.68	1.06	0.00	1.80	4.51	0.00	6.51	0.97	0.00	2.06
L VLDL	7.98	5.01	12.10	5.65	3.98	7.64	1.97	1.10	3.03	7.64	5.18	10.80	2.45	1.12	4.35
M VLDL	16.90	13.10	22.00	24.90	20.10	31.00	10.40	8.34	13.00	35.50	29.00	43.80	13.00	9.58	17.20
S VLDL	11.40	9.33	14.10	11.80	9.61	14.40	9.16	7.57	11.00	20.90	17.30	25.40	11.80	9.13	15.00
XS VLDL	5.94	5.04	7.05	17.80	15.20	21.10	5.48	4.71	6.33	23.20	20.00	27.50	8.33	6.97	9.67
Total IDL	9.19	7.74	10.60	63.30	54.80	73.40	22.20	19.40	25.50	85.90	74.20	99.10	29.30	25.80	33.50
L LDL	8.48	7.53	9.74	74.30	63.20	86.20	31.30	27.70	36.00	106.00	91.50	23.00	42.90	38.30	48.50
M LDL	3.73	3.33	4.35	24.30	21.00	28.20	14.40	12.30	16.70	38.70	33.20	44.80	20.50	17.80	23.40
S LDL	2.43	2.13	2.80	10.40	9.34	11.70	7.27	6.12	8.45	17.60	15.50	20.10	10.00	8.69	11.60
XL HDL	1.35	1.15	1.63	3.89	2.43	5.78	1.45	1.09	1.84	5.38	3.56	7.60	7.72	4.97	11.30
L HDL	3.93	3.12	4.78	20.20	13.70	29.60	3.19	2.19	4.57	23.40	15.90	34.20	33.30	24.40	44.80
M HDL	4.89	4.05	5.73	26.60	21.10	33.40	3.41	2.74	4.22	29.80	23.70	37.60	42.30	36.60	48.50
S HDL	5.97	5.07	6.89	34.30	30.20	38.30	4.64	4.16	5.15	38.90	34.30	43.20	45.10	40.60	50.10

^a^Concentration in M × 10^−5^

Abbreviations: *IQR* Inter quartile range, *XL* Extra large, *L* Large, *M* Medium, *S* Small, *XS* Extra Small, *VLDL* Very low density lipoprotein, *IDL* Intermediate density lipoprotein, *LDL* Low density lipoprotein, *HDL* High density lipoprotein

**Fig. 3:**
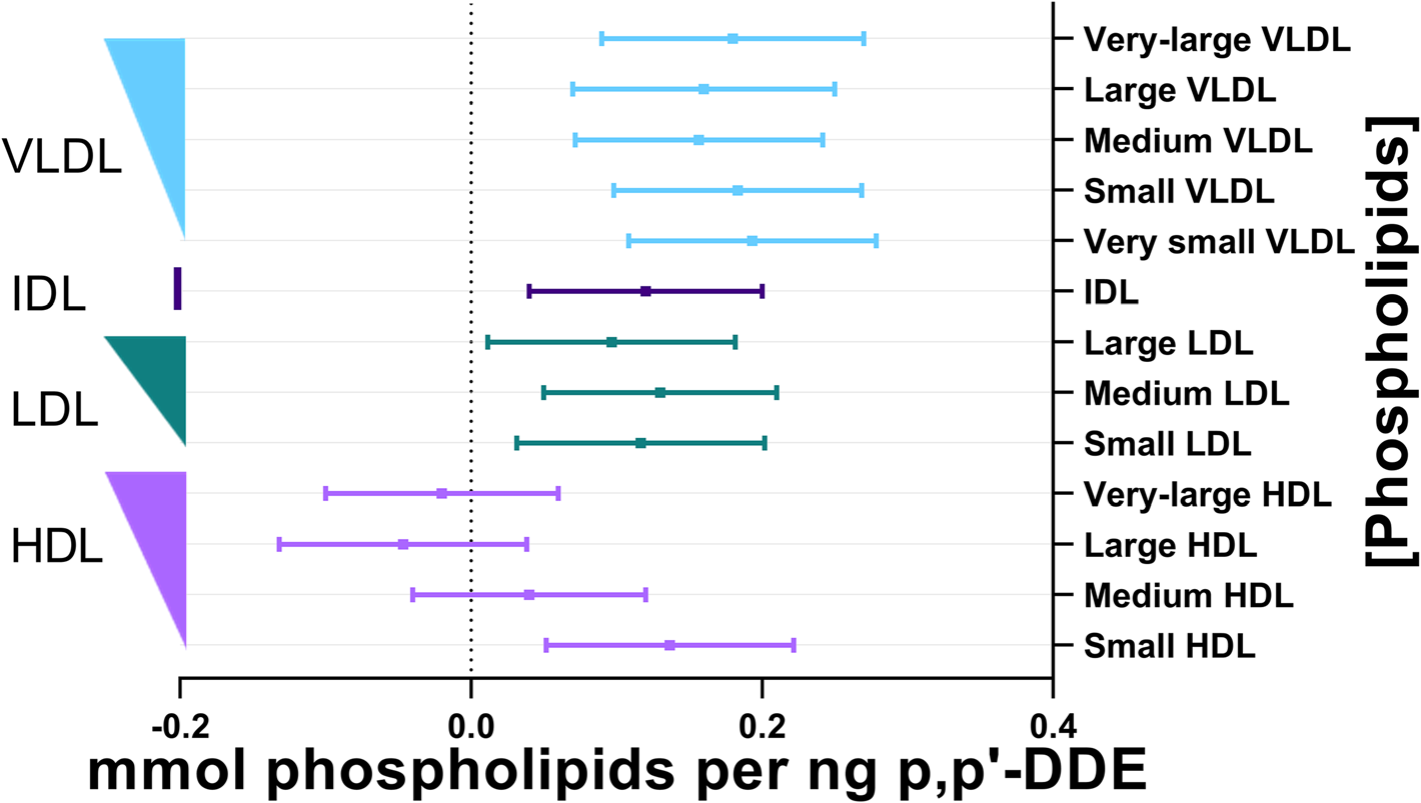
Association between plasma p,p’-DDE (ng) and phospholipids (mmol). The line of null effect is represented by the x-axis intercept and predicted margins are given together with 95% confidence intervals. The left y-axis illustrates the ordering of lipoprotein diameter in decreasing size, where the width of the right triangle corresponds to the relative diameter of the lipoprotein subclass

Similar to the associations observed between p,p’-DDE and triglycerides across lipoprotein fractions, p,p’-DDE and phospholipids had a significant positive association among all sizes of VLDL and LDL, in IDL, and in small HDL (Fig. 3).

**Fig. 4:**
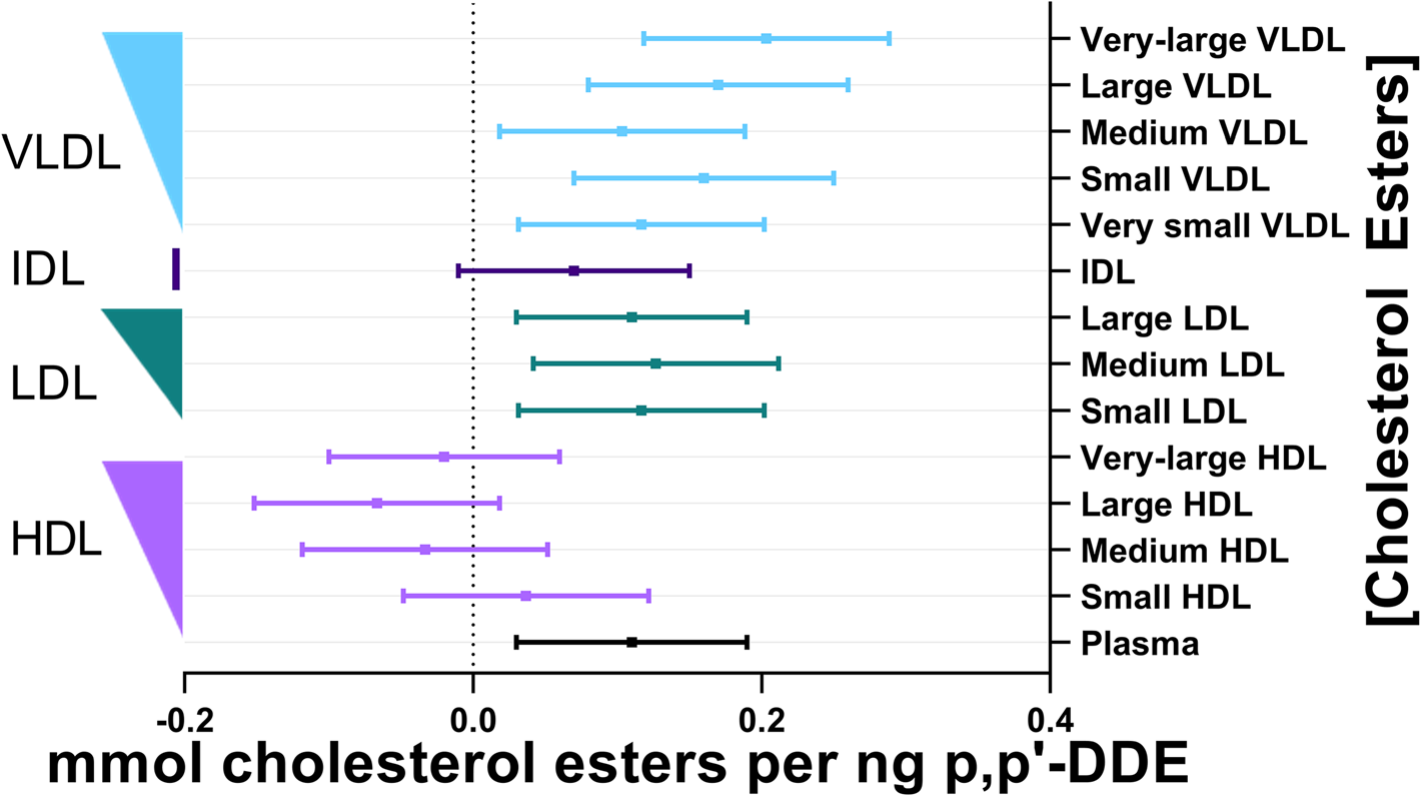
Association between plasma p,p’-DDE (ng) and cholesterol esters (mmol). The line of null effect is represented by the x-axis intercept and predicted margins are given together with 95% confidence intervals. The left y-axis illustrates the ordering of lipoprotein diameter in decreasing size, where the width of the right triangle corresponds to the relative diameter of the lipoprotein subclass

There was a similar magnitude of association between p,p’-DDE and total cholesterol, free cholesterol, and cholesterol esters within plasma (Supplementary Fig. 2., Figs. 3-4). p,p’-DDE was not significantly associated with total-, free-, or esters of- cholesterol found in IDL or in most sizes of HDL. The exception was the positive significant association between p,p’-DDE and free cholesterol in the small HDL fraction (Fig. 4.). p,p’-DDE was significantly associated with total-, free-, or esters of- cholesterol found in VLDL and in all LDL fractions but large LDL (Supplementary Fig. 2, Figs. 3-4). The significant associations of these cholesterols with p,p’-DDE had a similar magnitude across the different LDL sizes, and varied a bit more across VLDL sizes.

**Fig. 5:**
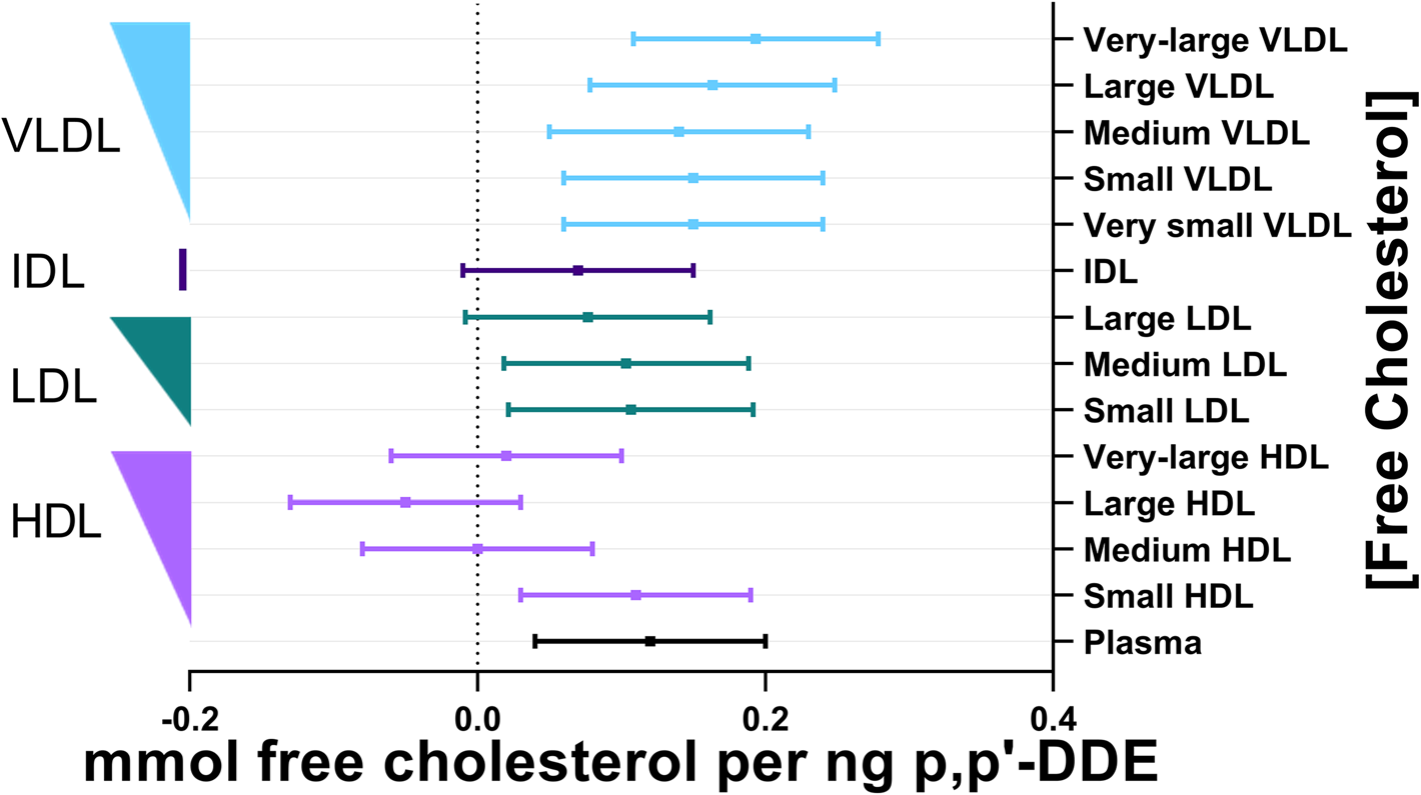
Association between plasma p,p’-DDE (ng) and free cholesterol (mmol). The line of null effect is represented by the x-axis intercept and predicted margins are given together with 95% confidence intervals. The left y-axis illustrates the ordering of lipoprotein diameter in decreasing size, where the width of the right triangle corresponds to the relative diameter of the lipoprotein subclass

It should also be noted that across total lipoprotein sizes and the lipid constituents of lipoproteins, the magnitude of associations with p,p’-DDE was most significant in VLDL, IDL, LDL, and small HDL, separating these classes from the larger diameters of HDL (Figs. 1, 2, 3, 4 and 5). There was no association of p,p’-DDE with any lipids found in the largest HDL particles (Figs. 1, 2, 3, 4 and 5).

Apolipoprotein B was significant in its positive association with p,p’-DDE (*P* < 0.001), driving the Apo B/Apo A1 ratio in the same direction (*P* < 0.05; Supplementary Fig. 3). Apolipoprotein A1 was not significantly associated with p,p’-DDE (*P* = 0.4; Supplementary Fig. 3).

## Discussion

Low density lipoproteins including VLDL, LDL, and IDL, and the lipids they carry have consistently been associated with CVD, suggesting that p,p’-DDE could play a role in exacerbating CVD through the positive association with this group of lipoproteins and their associated pathways [31–33].

Past research investigating the relationship between p,p’-DDE and lipoproteins has characterized sample sizes of fewer than 10 healthy human participants [25, 26]. For example, in a study of 5 fasted adults, p,p’-DDE was found to have the strongest association with LDL, followed by VLDL [25], wheras in this study p,p’-DDE exhibited the strongest association with VLDL, followed by LDL. This discrepancy between past results and those of the present study could be a product of the small sample sizes evaluated in previous studies [25, 26]. p,p’-DDE was also found to have the greatest association with the total abundance of VLDL and LDL in a study of 7 non-fasted adults, however VLDL and LDL were not separated as individual measurements, possibly obscuring the relationships [26].

Two major groupings of associations with p,p’-DDE were observed: 1) all diameters of VLDL, IDL, and LDL had a significant positive association with p,p’-DDE, and 2) the larger diameters of HDL had no significance with p,p’-DDE. These two groups biologically differ in their metabolic pathways. VLDL, IDL, and LDL are transported from the liver through the endogenous pathway to deposit lipids into other tissues. In contrast, HDL transports lipids from tissues such as the arteries through the reverse transport pathway to the liver [34]. It could be speculated that the difference in metabolic pathways explains the distinct associations of p,p;-DDE with these two groups of lipoproteins.

Associations between triglycerides and p,p’-DDE have been well studied and exhibit consistent results. Fluctuation in triglycerides following a meal proportionally increases serum levels of POPs, prompting the need for the normalization of POPs to total serum lipid content [35]. Even after factoring in the dynamic relationship between POP and triglycerides, significant associations between serum triglycerides and p,p’-DDE have been recorded in adults from the Native American community of Akwesasne Mohawks [36], China [37], and Europe [38, 39]. The positive association between p,p’-DDE and lipids was not uniform across the lipoprotein constituents, with triglycerides originating from lower density lipoproteins showing a similar magnitude of association with p,p’-DDE compared to triglycerides in high density lipoproteins. Within the PIVUS cohort, higher triglycerides have been associated with 15 cardiovascular-related protein biomarkers, suggesting a greater CVD risk with elevated triglyceride levels [40]. Other European cohorts have confirmed the association between high triglycerides and an increased risk of CVD [39, 41, 42].

Both cholesterol esters and free cholesterol were also positively associated with p,p’-DDE in the lower density lipoprotein classes. Total cholesterol associations with p,p’-DDE appear to be driven by cholesterol esters because the pattern of associations between p,p’-DDE and this summation were more similar to those of cholesterol esters, than free cholesterol. This finding supports previous data showing that cholesterol esters make up the majority of total cholesterol in lipoproteins [43]. Although this separation of cholesterol species could provide insight into other lipoprotein analyses, in this study there is little difference between each species, suggesting that total cholesterol is a sufficient measurement when assessing an association with p,p’-DDE. Furthermore, when total cholesterol is included with triglycerides in the equation used for the lipid normalization of POPs, the variability of POP concentration between fasted and fed state is significantly stabilized [44]. Even after lipid adjustment, a number of human cohorts have shown a significant association between total cholesterol and high levels of p,p’-DDE. Elevated cholesterol has been considered a predictor of CVD [45], particularly non-HDL cholesterol [46]. Within the PIVUS cohort, individual increases in LDL total cholesterol between age 70 and 75 were related to decreased flow-mediated vasodilation, a predictive factor in CVD risk [47]. Additionally, increased LDL total cholesterol in this cohort was associated with greater risk of increased intima-media thickness of the carotid artery, a factor used to determine plaque buildup in the arteries [48].

It is common to normalize p,p’-DDE levels for total lipids in circulation when evaluating exposure to POPs [11]. This was not performed in the present study because the aim was to explore how p,p’-DDE levels were linked to different lipid classes. The statistical models adjusted for BMI because obesity is a determinant of how much p,p’-DDE from the total body burden is stored in adipose tissue and this fact could influence the relationships between p,p’-DDE levels and different lipid classes.

### Study strength and limitations

A major strength of the present study is that multiple parameters, e.g. p,p’-DDE levels and the most extensive characterization of lipoproteins in a study of p,p’-DDE, were measured using a state of the art multiplatform approach. However not all lipoprotein size fractions, e.g. VLDL particles greater than 75 nm in diameter, were evaluated. Another strength of this study was the use of fasted blood samples in a cohort of over 500 people in a study of p,p’-DDE levels and lipids 100-fold larger than those performed in the past. While fasting is a routine control of confounding due to prandial state in modern analyses of lipids [49], most past studies of p,p’-DDE sampled p,p’-DDE and lipids without regard to prandial state. It must be acknowledged that PIVUS is an elderly, ethnically homogeneous cohort, limiting the ability to generalize results across other populations. This limitation could be mitigated by the replication of these novel associations between p,p’-DDE and lipid profiles within lipoproteins in epidemiological studies which include a more diverse range of ages and ethnicities.

## Conclusion

The positive relationship between p,p’-DDE levels and total concentration of lipoproteins, apolipoprotein B, and cholesterol found in this study resembles a prominent lipoprotein profile associated with CVD [50]. Thus, high exposure to p,p’-DDE could be an unrecognized risk factor for CVD, mediated by an atherogenic lipid profile. Further investigation into the role of p,p’-DDE in lipoprotein regulation is needed to fully understand the risk of p,p’-DDE exposure on cardiovascular health.

## Supplementary Information

**Additional file 1:** **Supplementary Table 1.** Association of p,p’-DDE level and lipoprotein concentration in PIVUS participants (*n* = 571). * mmol lipid per ng p,p’-DDE, adjusted for sex and BMI. † Bolded values indicate statistical significance (at *P-*values < 0.05). Abbreviations: *CI* Confidence interval, *VLDL* Very low density lipoprotein, *IDL* Intermediate density lipoprotein, *LDL* Low density lipoprotein, *HDL* High density lipoprotein.

**Additional file 2:** **Supplemental Fig. 1.** Association between plasma p,p’-DDE (ng) and total lipids, calculated as the sum of triglycerides, total cholesterol, and phospholipids, (mmol). The line of null effect is represented by the x-axis intercept and predicted margins are given together with 95% confidence intervals. The left y-axis illustrates the ordering of lipoprotein diameter in decreasing size, where the width of the right triangle corresponds to the relative diameter of the lipoprotein subclass.

**Additional file 3:** **Supplemental Fig. 2.** Association between plasma p,p’-DDE (ng) and total cholesterol, the sum of cholesterol esters and free cholesterol, (mmol). The line of null effect is represented by the x-axis intercept and predicted margins are given together with 95% confidence intervals. The left y-axis illustrates the ordering of lipoprotein diameter in decreasing size, where the width of the right triangle corresponds to the relative diameter of the lipoprotein subclass.

**Additional file 4:** **Supplemental Fig. 3.** Association between plasma p,p’-DDE (ng) and apolipoproteins (g). The line of null effect is represented by the x-axis intercept and predicted margins are given together with 95% confidence intervals.

## Data Availability

The datasets during and/or analyzed during the current study are available from the corresponding author on reasonable request.

## Abbreviations

BMI: Body mass index
CVD: Cardiovascular disease
CE: Cholesterol esters
p,p’-DDE: Dichlorodiphenyldichloroethylene
DDT: Dichlorodiphenyltrichloroethane
FC: Free cholesterol
HDL: High density lipoprotein
HPLC: High-performance liquid chromatography
IDL: Intermediate density lipoprotein
LDL: Low density lipoprotein
NIST: National Institute for Standards and Technology
POP: Persistent organic pollutant
PL: Phospholipids
PIVUS: Prospective Study of Vasculature in Uppsala Seniors
^1^H NMR: Proton nuclear magnetic resonance
QC: Quality control
C: Total cholesterol
L: Total lipids
TG: Triglycerides
VLDL: Very low density lipoprotein
