# Supplementary material for: The associations between p,p’-DDE levels and plasma levels of lipoproteins and their subclasses in an elderly population determined by analysis of lipoprotein content
Source: Lipids Health Dis. 2020 Dec 7;19:249. doi: 10.1186/s12944-020-01417-1 (PMC7722417; doi:10.1186/s12944-020-01417-1)
Supplement: Supplementary file 1 — Additional file 1: Supplementary Table 1. Association of p,p’-DDE level and lipoprotein concentration in PIVUS participants (n = 571). * mmol lipid per ng p,p’-DDE, adjusted for sex and BMI. † Bolded values indicate statistical significance (at P-values < 0.05). Abbreviations: CI Confidence interval, VLDL Very low density lipoprotein, IDL Intermediate density lipoprotein, LDL Low density lipoprotein, HDL High density lipoprotein. [file 12944_2020_1417_MOESM1_ESM.docx]

| **Metabolite** | **β**^*^ | **95% CI** | | ***P*-value** ^†^ |
| --- | --- | --- | --- | --- |
| HDL triglycerides | 0.13 | 0.04 | 0.22 | **0.0033682** |
| HDL cholesterol | -0.03 | -0.11 | 0.05 | 0.4640702 |
| Very large HDL total concentration | -0.02 | -0.10 | 0.06 | 0.6163369 |
| Very large HDL triglycerides | 0.05 | -0.04 | 0.13 | 0.2592622 |
| Very-large HDL phospholipids | -0.02 | -0.10 | 0.06 | 0.6079862 |
| Very large HDL lipids | -0.01 | -0.10 | 0.07 | 0.7271212 |
| Very large HDL free cholesterol | 0.02 | -0.06 | 0.10 | 0.6508489 |
| Very large HDL cholesterol esters | -0.02 | -0.10 | 0.06 | 0.6832764 |
| Very large HDL cholesterol | -0.01 | -0.09 | 0.07 | 0.8033398 |
| Large HDL total concentration | -0.05 | -0.13 | 0.03 | 0.2224946 |
| Large HDL triglycerides | 0.03 | -0.06 | 0.11 | 0.5375755 |
| Large HDL phospholipids | -0.05 | -0.13 | 0.04 | 0.2791295 |
| Large HDL lipids | -0.05 | -0.13 | 0.03 | 0.2174556 |
| Large HDL free cholesterol | -0.05 | -0.13 | 0.03 | 0.2143357 |
| Large HDL cholesterol esters | -0.07 | -0.15 | 0.02 | 0.1149839 |
| Large HDL cholesterol | -0.06 | -0.15 | 0.02 | 0.1241800 |
| Medium HDL total concentration | 0.01 | -0.07 | 0.09 | 0.8438106 |
| Medium HDL triglycerides | 0.15 | 0.07 | 0.24 | **0.0005967** |
| Medium HDL phospholipids | 0.04 | -0.04 | 0.12 | 0.3554869 |
| Medium HDL lipids | 0.01 | -0.07 | 0.09 | 0.7682112 |
| Medium HDL free cholesterol | 0.00 | -0.08 | 0.08 | 0.9480863 |
| Medium HDL cholesterol esters | -0.03 | -0.12 | 0.05 | 0.4181985 |
| Medium HDL cholesterol | -0.03 | -0.11 | 0.05 | 0.4766527 |
| Small HDL total concentration | 0.10 | 0.01 | 0.18 | **0.0222230** |
| Small HDL triglycerides | 0.17 | 0.08 | 0.26 | **0.0001358** |
| Small HDL phospholipids | 0.14 | 0.05 | 0.22 | **0.0012107** |
| Small HDL lipids | 0.11 | 0.03 | 0.19 | **0.0106219** |
| Small HDL free cholesterol | 0.11 | 0.03 | 0.19 | **0.0093150** |
| Small HDL cholesterol esters | 0.04 | -0.05 | 0.12 | 0.3862712 |
| Small HDL cholesterol | 0.04 | -0.04 | 0.13 | 0.2863497 |
| IDL total concentration | 0.10 | 0.02 | 0.18 | **0.0205747** |
| IDL triglycerides | 0.18 | 0.09 | 0.28 | **0.0000918** |
| IDL phospholipids | 0.12 | 0.04 | 0.20 | **0.0039776** |
| IDL lipids | 0.10 | 0.01 | 0.18 | **0.0222117** |
| IDL free cholesterol | 0.07 | -0.01 | 0.15 | 0.0959586 |
| IDL cholesterol esters | 0.07 | -0.01 | 0.15 | 0.1085699 |
| IDL cholesterol | 0.07 | -0.01 | 0.15 | 0.1011732 |
| LDL triglycerides | 0.17 | 0.08 | 0.26 | **0.0003850** |
| LDL cholesterol | 0.11 | 0.03 | 0.19 | **0.0108621** |
| Large LDL total concentration | 0.11 | 0.03 | 0.20 | **0.0085547** |
| Large LDL triglycerides | 0.16 | 0.07 | 0.26 | **0.0006400** |
| Large LDL phospholipids | 0.10 | 0.01 | 0.18 | **0.0232130** |
| Large LDL lipids | 0.11 | 0.03 | 0.19 | **0.0108041** |
| Large LDL free cholesterol | 0.08 | -0.01 | 0.16 | **0.0701928** |
| Large LDL cholesterol esters | 0.11 | 0.03 | 0.19 | **0.0094120** |
| Large LDL cholesterol | 0.10 | 0.02 | 0.19 | **0.0166671** |
| Medium LDL total concentration | 0.13 | 0.05 | 0.22 | **0.0027671** |
| Medium LDL triglycerides | 0.16 | 0.07 | 0.25 | **0.0006332** |
| Medium LDL phospholipids | 0.13 | 0.05 | 0.21 | **0.0026094** |
| Medium LDL lipids | 0.13 | 0.05 | 0.21 | **0.0026842** |
| Medium LDL free cholesterol | 0.10 | 0.02 | 0.19 | **0.0170472** |
| Medium LDL cholesterol esters | 0.13 | 0.04 | 0.21 | **0.0032885** |
| Medium LDL cholesterol | 0.12 | 0.04 | 0.21 | **0.0055734** |
| Small LDL total concentration | 0.13 | 0.04 | 0.21 | **0.0033908** |
| Small LDL triglycerides | 0.17 | 0.08 | 0.26 | **0.0003229** |
| Small LDL phospholipids | 0.12 | 0.03 | 0.20 | **0.0067020** |
| Small LDL lipids | 0.13 | 0.04 | 0.21 | **0.0037623** |
| Small LDL free cholesterol | 0.11 | 0.02 | 0.19 | **0.0123835** |
| Small LDL cholesterol esters | 0.12 | 0.03 | 0.20 | **0.0072233** |
| Small LDL cholesterol | 0.11 | 0.03 | 0.20 | **0.0078976** |
| VLDL triglycerides | 0.17 | 0.09 | 0.26 | **0.0000936** |
| VLDL cholesterol | 0.17 | 0.08 | 0.26 | **0.0002240** |
| Very large VLDL total concentration | 0.18 | 0.09 | 0.26 | **0.0000616** |
| Very large VLDL triglycerides | 0.15 | 0.07 | 0.24 | **0.0004153** |
| Very large VLDL phospholipids | 0.18 | 0.09 | 0.27 | **0.0000815** |
| Very large VLDL lipids | 0.18 | 0.10 | 0.27 | **0.0000307** |
| Very large VLDL free cholesterol | 0.19 | 0.11 | 0.28 | **0.0000164** |
| Very large VLDL cholesterol esters | 0.20 | 0.12 | 0.29 | **0.0000075** |
| Very large VLDL cholesterol | 0.20 | 0.12 | 0.29 | **0.0000072** |
| Large VLDL total concentration | 0.15 | 0.06 | 0.24 | **0.0006343** |
| Large VLDL triglycerides | 0.13 | 0.05 | 0.22 | **0.0019624** |
| Large VLDL phospholipids | 0.16 | 0.07 | 0.25 | **0.0003178** |
| Large VLDL lipids | 0.16 | 0.07 | 0.24 | **0.0004108** |
| Large VLDL free cholesterol | 0.16 | 0.08 | 0.25 | **0.0002669** |
| Large VLDL cholesterol esters | 0.17 | 0.08 | 0.26 | **0.0002279** |
| Large VLDL cholesterol | 0.17 | 0.08 | 0.26 | **0.0001784** |
| Medium VLDL total concentration | 0.15 | 0.06 | 0.23 | **0.0012239** |
| Medium VLDL triglycerides | 0.13 | 0.05 | 0.22 | **0.0028662** |
| Medium VLDL phospholipids | 0.16 | 0.07 | 0.24 | **0.0004767** |
| Medium VLDL lipids | 0.14 | 0.06 | 0.23 | **0.0013154** |
| Medium VLDL free cholesterol | 0.14 | 0.05 | 0.23 | **0.0022292** |
| Medium VLDL cholesterol esters | 0.10 | 0.02 | 0.19 | **0.0180487** |
| Medium VLDL cholesterol | 0.12 | 0.03 | 0.20 | **0.0093646** |
| Small VLDL total concentration | 0.18 | 0.09 | 0.27 | **0.0001078** |
| Small VLDL triglycerides | 0.14 | 0.06 | 0.23 | **0.0014091** |
| Small VLDL phospholipids | 0.18 | 0.1 | 0.27 | **0.0000454** |
| Small VLDL lipids | 0.18 | 0.09 | 0.27 | **0.0000909** |
| Small VLDL free cholesterol | 0.15 | 0.06 | 0.24 | **0.0011273** |
| Small VLDL cholesterol esters | 0.16 | 0.07 | 0.25 | **0.0005743** |
| Small VLDL cholesterol | 0.16 | 0.07 | 0.25 | **0.0006332** |
| Very small VLDL total concentration | 0.16 | 0.07 | 0.25 | **0.0004309** |
| Very small VLDL triglycerides | 0.17 | 0.08 | 0.27 | **0.0002448** |
| Very small VLDL phospholipids | 0.19 | 0.11 | 0.28 | **0.0000143** |
| Very small VLDL lipids | 0.17 | 0.08 | 0.25 | **0.0002361** |
| Very small VLDL free cholesterol | 0.15 | 0.06 | 0.24 | **0.0010637** |
| Very small VLDL cholesterol esters | 0.12 | 0.03 | 0.20 | **0.0078428** |
| Very small VLDL cholesterol | 0.13 | 0.04 | 0.21 | **0.0043655** |
| Apolipoprotein A1 | 0.03 | -0.05 | 0.11 | 0.4131631 |
| Apolipoprotein B | 0.15 | 0.06 | 0.23 | **0.0009359** |
| ApoB/ApoA1-ratio | 0.12 | 0.03 | 0.21 | **0.0105088** |
