# Supplementary figures and images for: The associations between p,p’-DDE levels and plasma levels of lipoproteins and their subclasses in an elderly population determined by analysis of lipoprotein content
Source: Lipids Health Dis. 2020 Dec 7;19:249. doi: 10.1186/s12944-020-01417-1 (PMC7722417; doi:10.1186/s12944-020-01417-1)

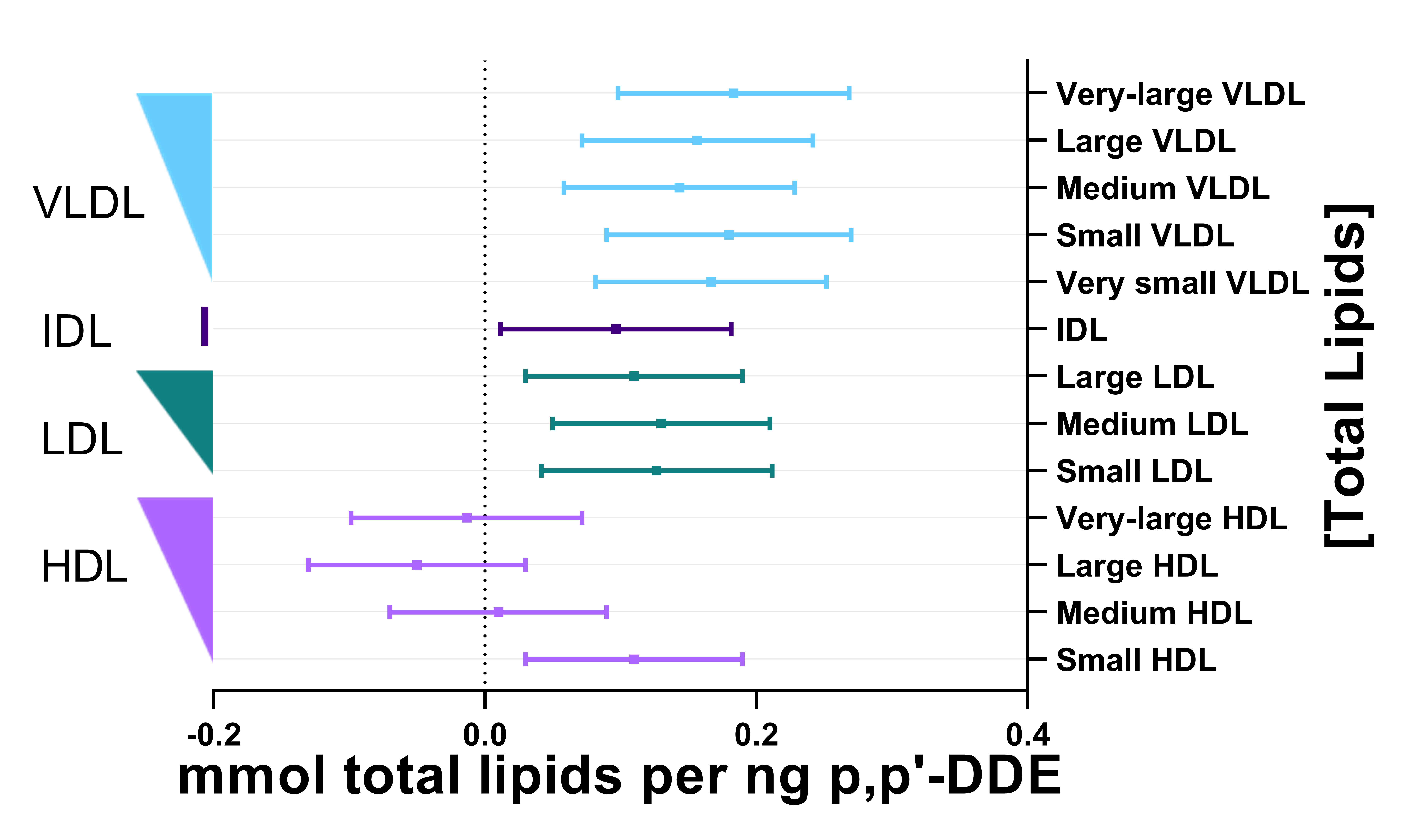

Supplement: Supplementary file 2 — Additional file 2: Supplemental Fig. 1. Association between plasma p,p’-DDE (ng) and total lipids, calculated as the sum of triglycerides, total cholesterol, and phospholipids, (mmol). The line of null effect is represented by the x-axis intercept and predicted margins are given together with 95% confidence intervals. The left y-axis illustrates the ordering of lipoprotein diameter in decreasing size, where the width of the right triangle corresponds to the relative diameter of the lipoprotein subclass. [file 12944_2020_1417_MOESM2_ESM.tif]

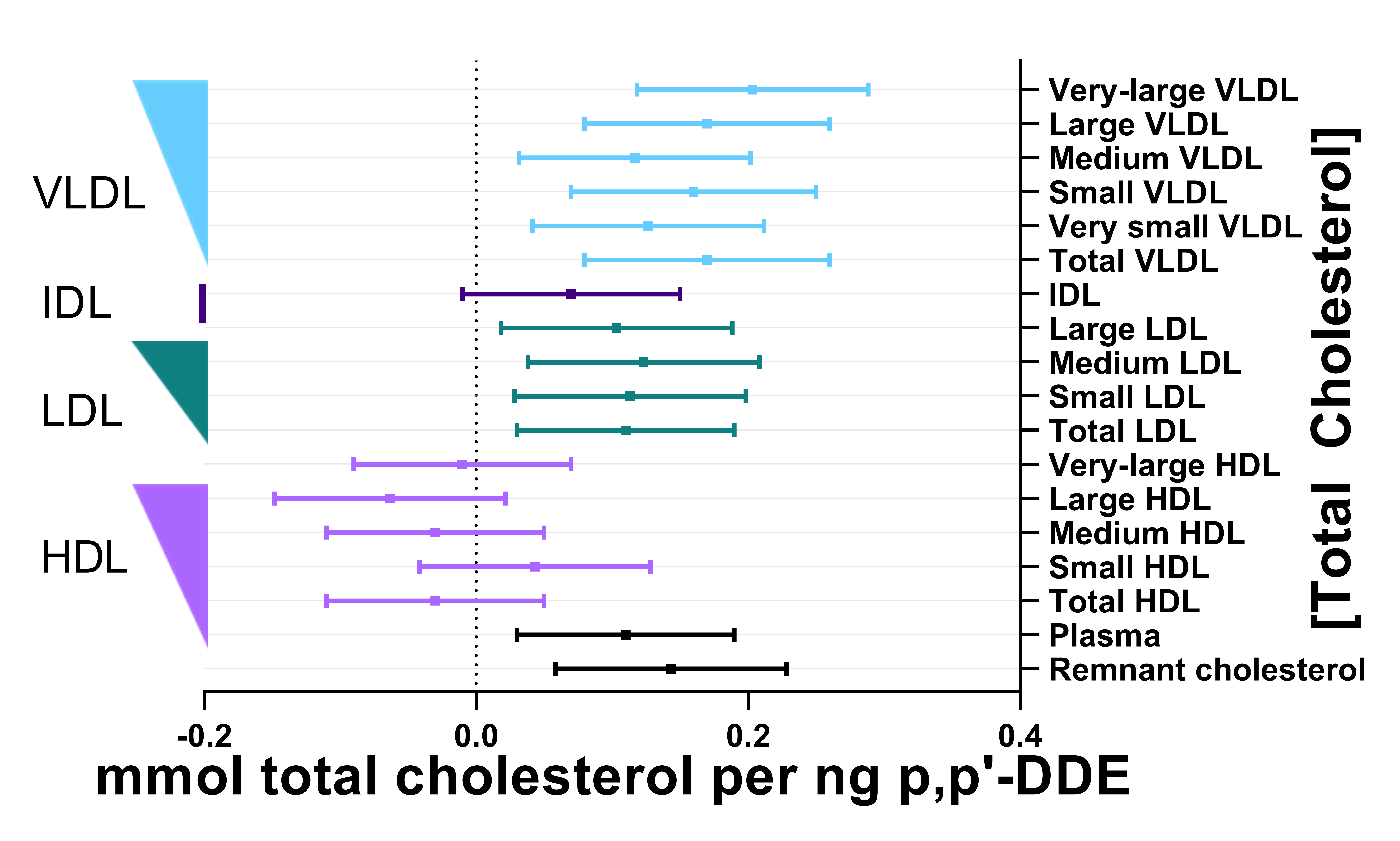

Supplement: Supplementary file 3 — Additional file 3: Supplemental Fig. 2. Association between plasma p,p’-DDE (ng) and total cholesterol, the sum of cholesterol esters and free cholesterol, (mmol). The line of null effect is represented by the x-axis intercept and predicted margins are given together with 95% confidence intervals. The left y-axis illustrates the ordering of lipoprotein diameter in decreasing size, where the width of the right triangle corresponds to the relative diameter of the lipoprotein subclass. [file 12944_2020_1417_MOESM3_ESM.tif]

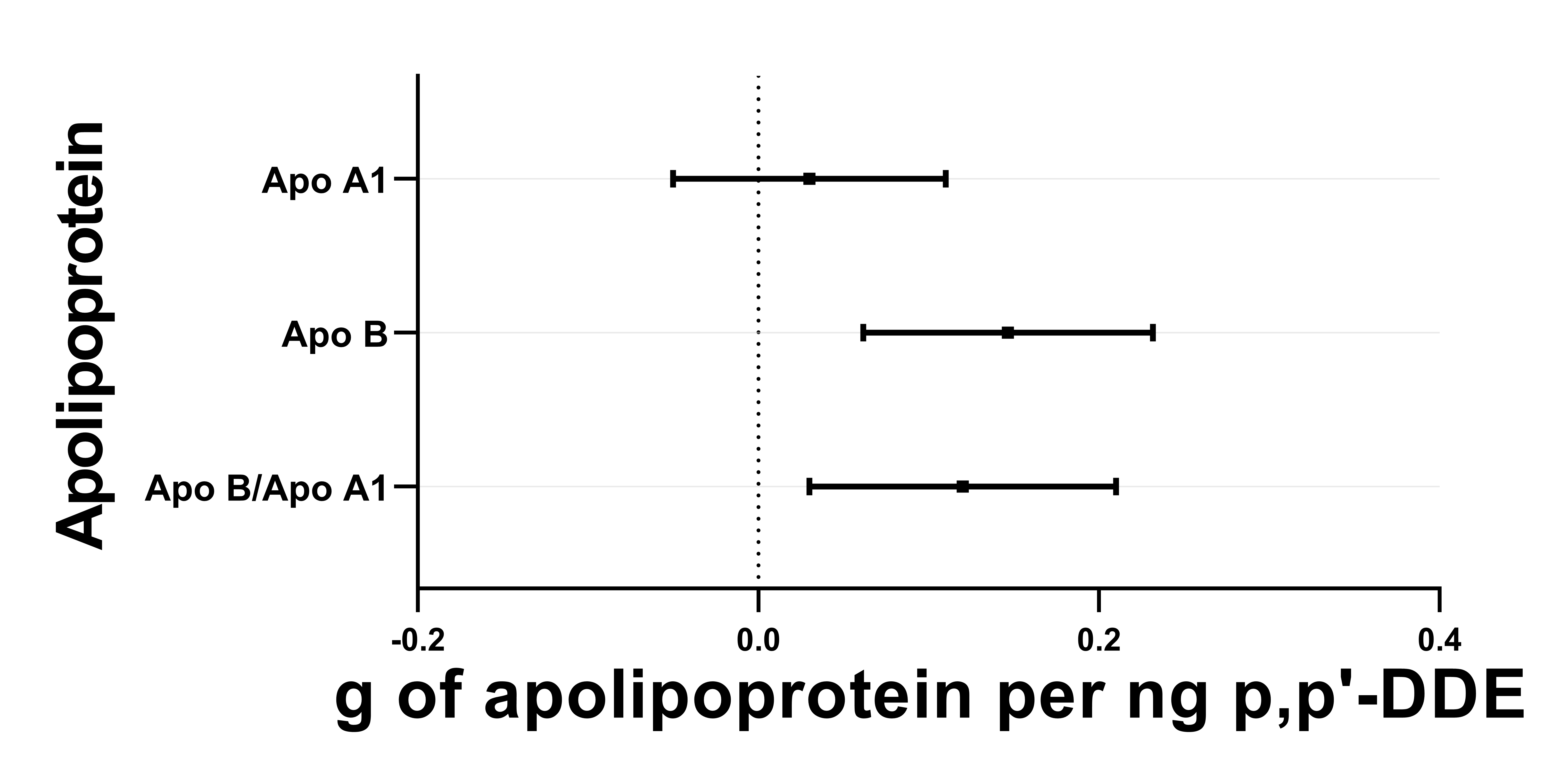

Supplement: Supplementary file 4 — Additional file 4: Supplemental Fig. 3. Association between plasma p,p’-DDE (ng) and apolipoproteins (g). The line of null effect is represented by the x-axis intercept and predicted margins are given together with 95% confidence intervals. [file 12944_2020_1417_MOESM4_ESM.tif]
